# Association between Expanded Disability Status Scale score and dietary antioxidant capacity in patients with multiple sclerosis

**DOI:** 10.1590/1414-431X2023e12776

**Published:** 2023-09-08

**Authors:** S. Mungan, I. Guzel, B.C. Demirdogen

**Affiliations:** 1 Department of Neurology, University of Health Sciences Ankara City Hospital, Ankara, Turkey; 2 Department of Neurology, Private Hatem Hospital, Gaziantep, Turkey; 3 Department of Biomedical Engineering, TOBB University of Economics and Technology, Ankara, Turkey

**Keywords:** Multiple sclerosis, Expanded Disability Status Scale, Antioxidant capacity

## Abstract

Multiple sclerosis (MS), a neuroinflammation that results in neurodegeneration, is the most prevalent central nervous system inflammatory disease in young people. A diet rich in antioxidants is known to decrease the production/activity of pro-inflammatory cytokines and have a positive impact on the prognosis of MS. The purpose of this study was to assess if dietary antioxidant capacity is related to Expanded Disability Status Scale (EDSS) scores in patients with MS. Patients with MS (n=220; 137 women and 83 men) were asked to complete a questionnaire on diet. According to the EDSS score, patients were split into two groups (group 1: EDSS ≤5 and group 2: EDSS >5). Analyzed risk variables were antioxidant levels and demographic data. A nutritional database tool (BeBiS 4 software, Germany) created for the evaluation of Turkish foods was used to examine the questionnaire findings. Age, vitamin A, retinol, vitamin D, vitamin E, and vitamin C were significantly different between groups (P<0.05). The levels of vitamins A, D, E, C, and retinol were significantly correlated, according to Pearson's correlation analysis. Receiver operator characteristic curve analysis revealed that vitamin A, vitamin D, and vitamin C levels were discriminating variables in group 2 patients (EDSS >5). The current study has shown that antioxidant levels obtained by EDSS may be useful in determining illness severity and treatment success of patients with MS. Further clinical trials have been initiated in MS patients with more effective antioxidants.

## Introduction

Multiple sclerosis (MS) is a complex inflammatory disease of the central nervous system with an autoimmune and chronic course and as yet unknown etiology. It is controversial whether inflammation triggers the characteristic neurodegeneration during MS pathogenesis or whether neurodegeneration develops independently of inflammation (1,2). This inflammatory response involves an interplay of B cells, T cells, macrophages, endothelial cells, and dendritic cells, as well as a number of inflammatory mediators, cytokines, chemokines, and reactive nitrogen and oxygen species (RNS and ROS) (3).

Based on epidemiological studies, several factors have been associated with disease development, such as low vitamin D status, certain viral infections (e.g., Epstein-Barr virus), childhood obesity, and dietary habits such as the so-called Western diet, which is characterized by high levels of saturated fat, carbohydrates, sodium chloride, and low fiber intake (4). Dietary habits have a direct impact on the composition of the gut microbiome, which affects the gut microbiota and thus the autoimmune responses of the whole body. Berer et al. (5) demonstrated that there is a direct link between gut microbiome and neuroinflammation. In their experimental study, they found that in the absence of commensal bacteria, encephalomyelitis can occur.

Previous studies have reported that poor dietary habits, unfavorable lifestyle, low vitamin D status, childhood obesity, or smoking may play a role in the progression of MS (6-9). The relationship between dietary factors and the pathogenesis of MS also remains unclear, and the impact of nutritional status on the prognosis of patients with MS has not been investigated.

The prognosis of MS was assessed using the Expanded Disability Status Scale (EDSS), a 20-point scale of disease severity ranging from 0 (normal) to 10 (death by MS) (10). In this study, we conducted a prospective survey of the diet of patients with MS to clarify the possible relationship between dietary antioxidant capacity and EDSS scores in patients with MS.

## Material and Methods

### Ethical Statement

This study was approved by the Research Ethics Committee of the Ankara City Hospital. The procedures applied in this investigation were in accordance with the Helsinki Declaration, and all study participants provided written informed consent.

### Study design and participants

This was a prospective study of multiple sclerosis cases conducted between January 1, 2019, and December 31, 2020, at Ankara City Research and Education Hospital, Turkey. The study included 220 patients with MS, of whom 137 were female and 83 were male.

Eligible subjects were patients with MS, nonsmoking Turkish adults between the ages of 21 and 69 years who were enrolled in our current education and research hospital. Eligibility requirements included a stable weight over the past 6 months and a willingness to maintain current dietary and exercise habits for the duration of the study. Subjects were excluded if any of the following conditions were present: gastrointestinal surgery, bowel disease, pancreatic disease, diabetes, hemophilia, alcoholism, mental disorders, hypothyroidism, bipolar disorder or seizure disorder, congestive heart failure, renal failure or other conditions affecting fluid balance, and current treatment with supplemental oxygen, antiretroviral drugs, antineoplastic drugs, anti-ulcer/antireflux drugs, or central nervous system drugs.

MS diagnosis was made according to the revised McDonald criteria (11). Patients were divided into two groups according to their EDSS score (group 1: EDSS ≤5; group 2: EDSS >5). Clinical data such as age, sex, body mass index, smoking, alcohol consumption, vitamin A, retinol, beta-carotene, vitamin D, vitamin E, vitamin B12, and vitamin C levels were collected from the study participants. Patients were informed about the study upon admission to our institution. The new 92-question antioxidant nutrient questionnaire from Satia et al. (12) was used to calculate dietary antioxidant capacity. The reviewed nutrient values were assessed after the survey results were entered into the nutrition database program (BeBiS 4 software; Bebispro, Germany) developed for the assessment of Turkish and commercial foods (13). Levels of vitamin A, retinol, beta-carotene, vitamin D, vitamin E, vitamin B12, and vitamin C were obtained from the questionnaire and calculated using the BeBis software. Subjects provided habitual food consumption information for the prior year using the 36-page, paper-based National Cancer Institute dietary-history food-frequency questionnaire (NCI-DHQ), which estimates portion sizes and frequency of consumption of 124 food items and also includes additional queries regarding specifications (e.g., fat content and seasonal consumption) for selected foods and supplement use over the past year.

The exclusion criteria were: initiation or change of immunomodulatory therapy within 6 months, a MS seizure (relapse) or cortisone treatment within 30 days before study entry, clinically significant progressive, metabolic, or malignant disease, intake of dietary supplements containing more than one gram of omega-3 fatty acids daily, significant impairment of cognitive-cooperative function, type I diabetes mellitus (insulin-dependent), participation in another intervention study, attempting to lose weight by dieting or weight loss of more than 5 kg within 2 months before study entry, lack of mental cooperative ability, eating disorders, pregnancy, breastfeeding, known drug or alcohol abuse, inability to sign an informed consent form or follow study protocols, contraindications to MRI examinations such as pacemakers, metallic implants, or claustrophobia.

### Statistical analysis

Continuous variables are reported as means±SD and their normality was tested using the Kolmogorov-Smirnov test. The independent-samples *t*-test was used to assess the differences between groups for continuous variables. The chi-squared test was used to compare categorical variables, which are presented as frequencies. Logistic regression analysis was carried out and all variables were included in the stepwise backward procedure. Receiver operator characteristic curve (ROC) analysis was performed to establish the cut-off values for vitamin A, vitamin D, and vitamin C concentrations. Two-tailed P values were considered statistically significant at P<0.05. Statistical analyses were performed using SPSS 16.0 for Windows (SPSS Inc., IBM, USA).

## Results

### Demographic characteristics

Table 1 summarizes the demographic characteristics and clinical information of the patients. Patients were split into two groups depending on their EDSS scores. Group 1 was composed of patients with EDSS ≤5 (n=166), and the mean age of patients was 35.1±9.7. In group 2, patients with EDSS >5 were included (n=54), and the mean age was 43.2±9.9 years (P<0.05). No statistically significant difference was observed between groups in terms of sex, smoking, and alcohol consumption (P>0.05).

**Table 1:** Demographic and clinical information of multiple sclerosis patients with Expanded Disability Status Scale (EDSS) score ≤5 and EDSS>5.

	Patients with EDSS ≤5(n=166)	Patients with EDSS>5(n=54)	P
Age (years)	35.1±9.7	43.2±9.9	0.023
Gender			0.232
Male (n,%)	63 (37.9)	20 (37.1)	
Female (n,%)	103 (62.1)	34 (62.9)	
Body mass index (kg/m^2^)	24.78±4.05	25.72±3.40	0.129
Current smoker (n,%)	78 (46.9)	46 (85.1)	0.423
Alcohol consumption (n,%)	34 (20.4)	38 (70.3)	0.622
Vitamin A (mg/L)	2.35±0.46	1.19±0.23	**0.016**
Retinol (mg/L)	2.52±0.60	0.64±0.28	**0.029**
Beta-carotene (μg/mL)	1.44 ±1.00	1.24±0.74	0.174
Vitamin D (ng/mL)	2.52±0.52	0.79±0.16	**0.018**
Vitamin E (μg/mL)	5.07±2.52	4.10±2.42	**0.014**
Vitamin B12 (pg/mL)	566.60±40.32	622.55±30.12	0.433
Vitamin C (mg/dL)	8.70±2.12	2.05±1.02	**0.009**

Data are reported as mean±SD. P-values in bold type are statistically significant (Student’s *t*-test and chi-squared test).

### Dietary supplements

Levels of dietary supplements are summarized in Table 1. Beta-carotene levels were 1.44±1.00 μg/mL in group 1 and 1.24±0.74 μg/mL in group 2 and vitamin B12 levels were 566.60±40.32 pg/mL in group 1 and 622.55±30.12 pg/mL in group 2. Both of these supplements were not significantly different between the groups (P>0.05).

The levels of vitamin A, retinol, vitamin D, vitamin E, and vitamin C were significantly higher in group 1 (P<0.05).

### Correlations between dietary intake and EDSS

The logistic regression analysis revealed a significant relationship between EDSS score and vitamin A, retinol, vitamin D, vitamin E, and vitamin C levels (Table 2). Lower levels of these vitamins were significant risk factors for higher EDSS (P<0.05).

**Table 2:** Association between Expanded Disability Status Scale (EDSS) score and dietary antioxidants.

	β	S.E.	Wald	Odds ratio	95%CI	P
Vitamin A	0.001	0.000	0.139	1.410	0.999-1.002	0.001
Retinol	0.001	0.002	0.864	0.999	0.995-1.002	0.002
Vitamin D	0.177	0.906	0.038	1.194	0.202-7.049	0.002
Vitamin E	4.441	1.792	6.138	2.786	0.283-27.418	0.012
Vitamin C	−5.175	2.141	5.841	1.635	0.001-2.042	0.032

Logistic regression was used for statistical analyses. S.E., standard error.

**Figure 1:**
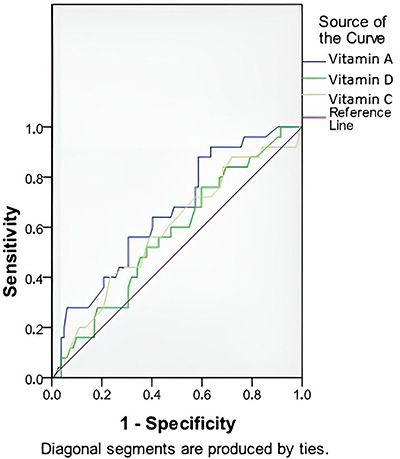
The receiver operator characteristic (ROC) curve for vitamin A, vitamin D, and vitamin C levels in patients with Expanded Disability Status Scale (EDSS) >5 in patients with multiple sclerosis.

The ROC curve analysis (Figure 1) showed that vitamin A, vitamin D, and vitamin C levels were discriminatory factors for EDSS ≤5 *vs* EDSS >5. The area under the curve (AUC) and P-values were 0.716 (P=0.012), 0.668 (P=0.024), and 0.617 (P=0.015), respectively.

## Discussion

In the current study, 220 patients with MS were invited to participate in a novel, self-administered 92-item antioxidant survey based on semiquantitative assessment of frequency of food intake (12). The groups differed significantly in age, vitamin C, vitamin A, retinol, vitamin E, and vitamin D (P<0.05).

The effect of antioxidants in neurologic diseases has been studied previously. Ghareghani et al. (14) examined the influence of vitamin D, melanopsin, pineal gland, intestinal calcium, intestinal endotoxins (lipopolysaccharides; LPS), and CD14/TLR4 in neurologic diseases, and found that low vitamin D levels and impaired intestinal calcium absorption could lead to migration of LPS to the brain and trigger the release of proinflammatory cytokines via the CD14/TLR4/MD2 complex. Similarly, in our study, we found that antioxidants were lower in patients with poor prognosis MS.

The role of some antioxidant compounds such as vitamin D3, melatonin, omega-3 polyunsaturated fatty acids, and polyphenolic substances as adjunctive therapy in multiple sclerosis was described in a review by Miller et al. (15). The authors found that the use of immunomodulatory drugs and supplementation with antioxidants reduced the production/activity of proinflammatory cytokines and may have a positive effect on the prognosis of MS. Bitarafan et al. (16) conducted a double-blind, placebo-controlled clinical trial in 101 patients with MS investigating the effect of vitamin A supplementation on psychiatric symptoms such as fatigue and depression. They showed that an antioxidant-rich diet improved fatigue and depression in MS patients by modulating inflammatory states. Our results are consistent with this finding, as we also found that vitamin A, vitamin D, and vitamin C levels were decreased in the group of patients with EDSS >5.

There is a growing body of evidence from randomized placebo-controlled trials and observational studies on the effects of antioxidant diets, ketogenic diets, and fasting diets. Hadžović-Džuvo et al. (17) performed a cross-sectional study and determined total antioxidant capacity in patients with MS. They showed that total antioxidant capacity was significantly decreased in patients with MS compared with the control group. In another study, Besler et al. (18) reported that retinol, alpha-tocopherol, beta-carotene, and ascorbic acid were significantly lower in the MS group than in the control group. In a controlled case study, total antioxidant status, glutathione peroxidase, and serum selenium levels of MS patients were significantly lower than the controls, and these parameters were associated with poor prognosis (19). Our results are consistent with these studies.

Felicetti et al. (20) also conducted a study to investigate the association between a Mediterranean diet, which has a beneficial effect in neurodegenerative diseases, and EDSS in MS patients. In a cross-sectional study by Coe et al. (21) and another study by Di Majo et al. (22), positive health outcomes were found with higher consumption of carotene, magnesium, fatty fish, fruits and vegetables, and sodium and worse outcomes with consumption of fiber, red meat, and saturated fat (women only). Similar to these articles, we found a positive effect of antioxidants on EDSS in MS patients.

In conclusion, the present study assessed the role of nonpharmacological dietary antioxidants in the prognosis of MS. Further studies with more participants are needed to identify new targets for innovative therapies to improve outcomes in patients with MS.
